# ABO blood group and the risk of placental malaria in sub-Saharan Africa

**DOI:** 10.1186/1475-2875-10-101

**Published:** 2011-04-22

**Authors:** Ayola A Adegnika, Adrian JF Luty, Martin P Grobusch, Michael Ramharter, Maria Yazdanbakhsh, Peter G Kremsner, Norbert G Schwarz

**Affiliations:** 1 Medical Research Unit, Albert Schweitzer Hospital, Lambaréné, Gabon; 2 Institute of Tropical Medicine, University of Tübingen, Tübingen, Germany; 3 Department of Parasitology, Leiden University Medical Center, Leiden, The Netherlands; 4 Infectious Diseases, Tropical Medicine and AIDS, Department of Internal Medicine, Academic Medical Center, University of Amsterdam, The Netherlands; 5 Department of Medicine I, Division of Infectious Diseases and Tropical Medicine, Medical University of Vienna, Austria; 6 Bernhard Nocht Institute for Tropical Medicine, Infectious Disease Epidemiology Bernhard-Nocht Straße 74D-20359 Hamburg, Germany

## Abstract

**Background:**

In malarious areas of the world, a higher proportion of the population has blood group O than in non-malarious areas. This is probably due to a survival advantage conferred either by an attenuating effect on the course of or reduction in the risk of infection by plasmodial parasites. Here, the association between ABO blood group and incidence of placental malaria was assessed in order to determine the possible influence of the former on the latter.

**Methods:**

Data from a study in Lambaréné, Gabon, and data from three previously published reports of studies in The Gambia, Malawi and Sudan, were compiled and compared. ABO blood groups were cross-tabulated with placental malaria stratified by parity. Odds ratios (OR), stratified by parity, were calculated for the outcome, placental parasitaemia, and compared between blood group O vs. non-O mothers in all four studies. Random effects meta-analysis of data from individual studies from areas with perennial hyper/holoendemic transmission was performed.

**Results:**

In Gabon, the odds ratio (OR) for active placental parasitaemia in mothers with group O was 0.3 (95% CI 0.05-1.8) for primiparae and 0.7 (95% CI 0.3-1.8) for multiparae. The OR for primiparae in the published study from The Gambia was 3.0 (95% CI 1.2-7.3) and, in Malawi, 2.2 (95% CI 1.1-4.3). In the Sudanese study, no OR for primiparae could be calculated. The OR for placental parasitaemia in group O multiparae was 0.8 (95% CI 0.3-1.7) in the Gambia, 0.6 (95% CI 0.4-1.0) in Malawi and 0.4 (95% CI 0.1-1.8) in Sudan. Combining data from the three studies conducted in hyper-/holo-endemic settings (Gambia, Malawi, Gabon) the OR for placental malaria in blood group O multiparae was 0.65 (95% CI 0.44-0.96) and for primiparae 1.70 (95% CI 0.67-4.33).

**Conclusion:**

Studies conducted in The Gambia and Malawi suggest that blood group O confers a higher risk of active placental infection in primiparae, but a significantly lower risk in multiparae. These findings were not confirmed by the study from Gabon, in which statistically non-significant trends for reduced risk of placental parasitaemia in those with blood group O, regardless of parity, were observed.

## Background

Since the Nobel Prize-rewarded discovery of the ABO blood group system in 1900 by Karl Landsteiner [1], many studies have examined the associations between these blood groups and either infectious or non-infectious diseases [2]. Associations between group A and gastric cancer and group O and lung cancer have been demonstrated [3,4]; such diseases, however, primarily affect humans who have surpassed their reproductive age and are therefore not expected to influence genetic selection. In contrast, the death toll from malaria is highest in children and plasmodial parasites invade erythrocytes, the very cells that express the ABO antigens on their surface. Malaria is thus a disease for which an association with ABO blood group distribution seems most plausible, and it may have played an important role in shaping the prevalence in favour of group O [5]. Supporting evidence for this hypothesis includes i) the worldwide distribution of the ABO blood groups with a type O predominance in malarious regions of the world [6]; ii) the fact that *Plasmodium falciparum *has substantially affected the human genome [7] and was present when the ABO polymorphisms arose [6], iii) the associations of ABO blood groups and clinical outcome of malaria, and iv) finally the pathophysiological plausibility of an interaction between the malaria parasite and the red blood cell and the potential role erythrocyte surface antigens may play in cytoadhesion of infected erythrocytes and parasite invasion. Numerous studies have been carried out on the association between the ABO blood groups and malaria [8], concluding that blood group O seems to confer a certain degree of protection against severe courses of the disease [9-12].

Apart from children, pregnant women are particularly susceptible to infections with *P. falciparum*. Such infections during pregnancy affect not only the mother, since accumulations of infected erythrocytes in the placenta hamper its function and consequently disturb foetal development. Adverse outcomes include low birthweight, prematurity or even stillbirth. The potential for selective pressure by *P. falciparum*, on the ABO blood groups as well as multiple other genetic loci, is thus clear. There are few published studies examining the association between ABO blood groups and placental malaria [13-15]. In recently published data from the Gambia and recent data from Malawi, blood group O was found to be significantly associated with increased placental malaria infection in primiparae and a reduced risk of placental malaria infection in multiparae [14,15]. In eastern Sudan, an association between blood group O and past placental infection was observed in primi- and in multiparae [13].

Here, using data from a study in Lambaréné, Gabon, the association between the ABO blood groups and active placental malaria was investigated and compared directly with the findings of the other studies published on this topic.

## Methods

Original data were obtained from a study of malaria in mother-child pairs that was conducted during the period 2003-2004 at the Albert Schweitzer Hospital in Lambaréné, Gabon. A comparative analysis was performed, using these data and three previously published studies, to assess the effect of blood group O versus non-O on the incidence of placental malaria in primi- and in multiparae.

### Study population and area for the original data collection

Lambaréné is located near to the equator in Central Africa in an area with perennial malaria transmission. Data are from a study on malaria and parasitic co-infections during pregnancy. Further information on this study was previously published [16,17]. For assessment of the infection status of consecutively enrolled mothers, placental blood was drawn immediately after delivery from the intervillous spaces at the centre of the placental tissue, and Giemsa-stained thick smears prepared. Parasitaemia was quantified using a standardized microscopical method as previously described [18]. ABO blood groups were typed by agglutination using commercially available antiserum-kits. Informed consent was sought and obtained from all mothers who were included in the study and additionally from their parents in the case of minors. The study was approved by the ethics committee of the International Foundation of the Albert Schweitzer Hospital.

### Data from other studies used for the comparative analysis

Data from the three published studies were taken from the original publications. To compare all 4 studies a dataset was constructed at the individual level (1 set per mother) consisting of a categorical variable for blood groups (A, B, AB, and O), a categorical variable indicating the source study [13-15] and binary variables for parity (primipara vs. multipara) and placental parasitaemia. The outcome variable "placental malaria" was subdivided into "acute infection", defined as the presence of parasites without pigment, "chronic infection", defined as the presence of parasites and pigment, and "past infection", defined as the presence of pigment without parasites. Acute and chronic placental malaria were grouped as "active infection" [14,19]. For the comparison performed here, this latter definition of active placental malaria was used. Therefore, in the comparison, the variable "placental parasitaemia" was positive if parasites were present either with (chronic infection) or without (acute infection) pigment. A second study from Sudan, carried out in close proximity to the first hospital-based study, was recently published [20], however it did not contain the detailed data necessary for comparison with the others. The main exposure variable of interest was ABO blood group type. Analyses were carried out using stratification by parity (primi- vs- multipara) to account for parity as a potential effect modifier or a confounder for placental malaria.

### Statistical methods

Statistical analyses were conducted using the software package "STATA 10". A combined dataset, using published data and data from our own study, was generated in Microsoft Excel. The chi-square or Fisher's exact test was used for significance testing of categorical variables and the Student's t-test for mean values of continuous variables. Odds ratios were calculated using logistic regression with respective 95% confidence intervals. A meta-analysis generating Forest plots was performed for those studies providing data from hyper-/holo-endemic settings with stable transmission using a random effects model.

## Results

### Study results from Lambaréné/Gabon

A total of 467 women were included in the study, but for the analyses performed here data from 91 women, for whom no placental blood samples had been taken and from two women for whom the information on the ABO blood group was missing, were excluded. Therefore the analytical population consisted of 378 mothers (mean age 24.8, SD 6.5) of whom 84 (22%) were primiparae. The distribution of the exposure (ABO blood groups) and the outcome (placental malaria) did not differ significantly between women who were excluded from the analysis compared to those who were included (Fisher's exact test p = 0.80). However the proportion of primiparae was slightly lower in the analysed population (22.2%) compared to those excluded (29.7%, Fisher's exact test p = 0.09, Pearson chi2 p = 0.13). The distribution of blood group types did not differ significantly in multi- compared to primiparae (p-chi2 = 0.88). The OR for placental parasitaemia in mothers with blood group O versus non-O was 0.6 (95% CI 0.3-1.3). When stratified by parity the respective ORs were 0.3 (95% CI 0.05-1.8) for primiparae and 0.7 (95% CI 0.3-1.8) for multiparae.

### Comparative analysis using data from the three previous studies and data from Gabon to assess the effect of blood group O compared to non-O on placental malaria

In The Gambia, the proportion of mothers with blood group O was 52%, whilst it was 49% in Malawi, 44% in Sudan and 63% in the present Gabonese study. Thus the proportion of mothers with blood group O significantly varies between the countries (Chi2 <0.0001). In the Gambia, 37.4% of all mothers had active placental parasitaemia, compared with 19.3% in Malawi, 4.1% in Sudan and 7.1% in Gabon. In The Gambia primiparae had a significantly higher probability of placental parasitaemia compared to multiparae (47% vs 30%, p = 0.016). None of the three more recent studies found a significant difference between primi- and multiparae in this respect. Data derived from the different studies are shown in Table 1.

**Table 1:** Placental malaria* and ABO phenotype by parity in different studies

		Primiparae			Multiparae			All		
	ABO	N	Plac. Pos.*	%	N	Plac. Pos.*	%	N	Plac. Pos.*	%
Loscertales et al.	A	16	6	37.5	22	6	27.3	38	12	31.6
Senga et al.	A	51	5	9.8	95	21	22.1	146	26	17.8
Adam et al.	A	31	2	6.5	51	5	9.8	82	7	8.5
Present study	A	18	3	16.7	59	2	3.4	77	5	6.5
Loscertales et al.	B	20	6	30.0	32	11	34.4	52	17	32.7
Senga et al.	B	50	11	22.0	111	22	19.8	161	33	20.5
Adam et al.	B	24	0	0	35	2	5.7	59	2	3.4
Present study	B	14	1	7.1	40	6	15.0	54	7	13.0
Loscertales et al.	AB	2	0	0	4	2	50.0	6	2	33.3
Senga et al.	AB	6	1	16.7	15	5	33.3	21	6	28.6
Adam et al.	AB	8	1	12.5	16	0	0	24	1	4.2
Present study	AB	2	0	0	7	1	14.3	9	1	11.1
Loscertales et al.	O	50	29	58.0	52	14	26.9	102	43	42.2
Senga et al.	O	96	28	29.1	213	30	14.1	309	58	18.8
Adam et al.	O	51	0	0	77	2	2.6	128	2	1.6
Present study	O	50	2	4.0	188	12	6.4	238	14	5.9
Loscertales et al.	All	88	41	46.6	110	33	30.0	198	74	37.4
Senga et al.	All	203	45	22.2	434	78	18.0	637	123	19.3
Adam et al.	All	114	3	2.6	179	9	5.0	293	12	4.1
Present study	All	84	6	7.1	294	21	7.1	378	27	7.1

*Acute infection (parasites present without pigment) or chronic infection (parasites present with pigment).

**Figure 1:**
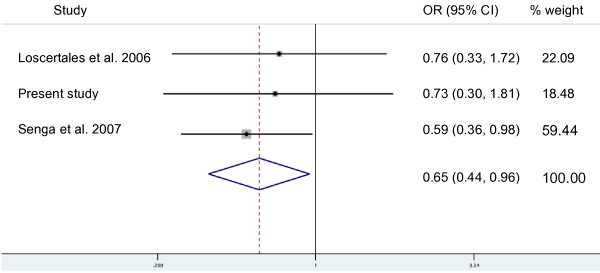
**Meta analysis of three different studies from holoendemic settings on the association between blood group O vs. other ABO blood groups and active placental infection in multiparae**. Note: The study by Adam *et al *[20] was excluded from the meta-analysis as no perennial transmission took place in the corresponding study site in eastern Sudan.

**Figure 2:**
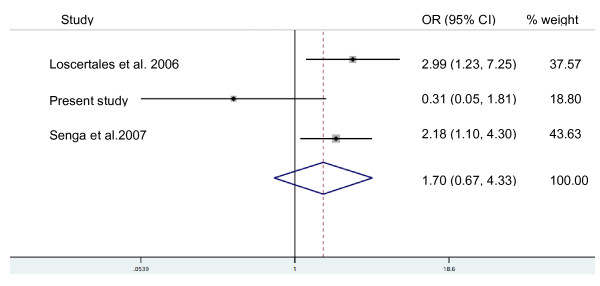
**Meta analysis of three different studies from holoendemic settings on the association between blood group O vs. other ABO blood groups and active placental infection in primiparae**. Note: The study by Adam *et al *[20] was excluded from the meta-analysis as no perennial transmission took place in the corresponding study site in eastern Sudan.

The OR stratified by parity for each study were calculated, and an overview of these is given in Table 2. For primiparae, two studies showed a clear positive association of blood group O and active placental parasitaemia (OR 3.0, 95% CI 1.2-7.3 & OR 2.2, 95% CI 1.1-4.3). This association was not reflected in the data from Gabon. In the study from Sudan, the OR could not be calculated for primiparae. In multiparae all studies showed a protective effect of blood group O against placental malaria, however only in the largest study (647 mothers) was this effect statistically significant [15]. A second study from Sudan that is listed in Table S1 (Additional file 1) was only published as a research note and therefore does not contain the data required for the analyses presented in Tables 1 and 2. Based on a random effect model, a forest plot for the three studies that were carried out in hyper-/holo-endemic regions with stable transmission (The Gambia, Malawi and Gabon) was constructed comparing mothers with blood group O to those with other blood groups with respect to the presence of active placental infection (Figure 1 and 2). As parity may be an effect modifier for the effect of ABO blood group types on placental malaria, the random effect model was run separately for primi- and multiparae. This meta-analysis suggests that blood group O offers some protection against placental malaria in multiparae (combined OR 0.65; 95% CI 0.44 - 0.96). In primiparae the opposite effect (blood group O associated with a higher risk for placental malaria) was noted without reaching statistical significance (combined OR 1.70; 95% CI 0.67 - 4.33). Table S1 (Additional file 1) finally, gives an overview of all studies that were considered regarding their location, design, objectives and findings.

**Table 2:** Comparing the blood group O vs. non-O regarding placental malaria infection for primi- and multiparae for four different studies

		Primiparae			Multiparae			Total		
		N	OR	95% CI	N	OR	95% CI	N	OR §	95% CI
Loscertales et al.	Non-O	38			58			96		
	O-group	50	3.0	1.2-7.3	52	0.8	0.3-1.7	102	*1.5*	*0.9-2.7*
Senga et al.	Non-O	107			221			328		
	O-group	96	2.2	1.1-4.3	213	0.6	0.4-1.0	309	*0.9*	*0.6-1.4*
Adam et al.	Non-O	63			102			165		
	O-group	51	#	#	77	0.4	0.1-1.8	128	*0.3*	*0.1-1.1*
Present study	Non-O	34			106			140		
	O-group	50	0.3	0.1-1.8	188	0.7	0.3-1.8	238	0.6	0.3-1.3

**§ **If the stratified ORs (for primi- and multiparae) indicate that parity modifies the effect of blood group O on the risk of placental malaria, the combined ORs (for All) are printed in *grey italics *as they should not be used for interpretation of the findings

**# **In the Adam study there was no active placental parasitaemia in primiparae with blood group O therefore the OR could not be calculated

## Discussion

The OR comparing mothers from Lambaréné with blood group O to non-O blood groups regarding placental malaria were 0.3 (95% CI 0.05-1.8) for primiparae and 0.7 (95% CI 0.3-1.8) for multiparae. These data thus suggest a tendency towards a protective effect of the blood group O against placental malaria infection regardless of parity; however this was not statistically significant. The higher risk of placental parasitaemia in primiparae with blood group O that was found in two previous studies could not be confirmed. The significant association of blood group O with placental malaria in primiparae and a reduced risk of placental infection in multiparae that was found in the recently published 1968 data from the Gambia [14] and a recent study from Malawi [15] were not confirmed by a study conducted in Sudan [13] or by the study from Gabon. Both these studies included a smaller sample size than the study from Malawi and may therefore have lacked the power to detect an effect. They were nevertheless both bigger than the older study from the Gambia that, however, was carried out when the malaria burden was still much higher than it is now.

## Limitations

While other studies used histopathology, the study from Gabon used placental blood smear. The sensitivity of histopathology for detection of placental infection is indeed acknowledged to be greater, although by precisely how much has not been assessed. Placental blood smear only allows assessment of active placental malaria (presence of parasites) and in contrast to histopathology cannot detect past infections. Con-sequently only the association of ABO blood group and active but not past placental infection was compared in this meta-analysis. In contrast to other studies, in Gabon there was no difference in parasite prevalence between primi- and multiparae, however it seems unlikely that the absence of a higher parasitaemia in primiparae could modify the effect of the analysed exposure (ABO blood group) on the outcome (placental parasitaemia), especially when keeping in mind that all analyses were kept stratified by primi- vs. multiparity. The results from Gabon should certainly be interpreted with caution as the statistical power was low with very wide confidence intervals. Given that only a few studies have been published on the association between ABO blood groups and placental malaria, the possibility of publication bias has also to be born in mind, i.e. other studies have remained unpublished because no "interesting" relationships between the ABO blood groups and placental malaria infection were found. If the severity of pregnancy-associated malaria affected the probability of delivering at a hospital, all studies may bear a selection bias: if blood group O is associated with less severe courses of disease, women with more severe courses may not deliver in hospital, but rather stay sick at home or even have premature labour at home. However an inverse effect would also be credible: women with more severe courses may have a higher probability of seeking medical support and therefore deliver in the hospital. To rule out this bias, longitudinal studies are needed that include women at the beginning of their pregnancy, assess their blood groups and follow them up until their delivery, thus also recording women for whom no placental sample is available. A limitation of meta analyses that build on exposure and outcome figures from published studies is the inability to adjust for further confounding factors such as maternal age or nutritional status as for such analyses one would require the corresponding raw datasets from all studies. At least information on the most important effect modifier - parity - could be used and all analyses were stratified by this factor.

## Interpretation and conclusions

In the four studies that were assessed [13-15], between 44% and 63% of mothers had blood group O, a comparatively but expectedly high proportion compared to populations from non-malarious regions in which ~36% carry this blood group type. This is consistent with a survival advantage of group O for individuals exposed to malaria, although the degree of penetration indicates that non-O types must have some benefit with respect to other pathogens as they have not been completely lost [21].

There is conflicting evidence regarding the impact of ABO blood groups on the probability of malaria infections of the peripheral blood in non-pregnant individuals [22-27]. However there seems to be a relative resistance of blood group O-individuals to severe courses of the disease [9-12,28] (for a summary see the review by Uneke [8]).

In theory, it is conceivable that selective pressure either acts via a blood group- dependent differential risk of malaria or via a different survival probability when infected. Previous studies found associations between blood group O and relatively mild courses of malaria [9-12] thus indicating a selective pressure acting via a blood group-dependent differential survival probability. Under the assumption that blood group O additionally conferred its survival advantage by reducing the risk of infection of human beings and blood cells and not by modifying the course of the disease, one would expect a protective effect of blood group O against placental malaria infection. In the four studies that were compared here, three of them in a meta-analysis, some evidence for such a reduced risk of infection conferred by blood group O could be demonstrated in multiparae.

The hypothesis that the survival advantage of blood group O with respect to the threat malaria poses is probably rather due to a milder course of the disease than due to a lower risk of infection is supported by findings showing that the ABO locus is associated with the serum levels of molecules known to bind to *P. falciparum*-infected red blood cells that are also markers of damage to vascular endothelial cells and inflammatory processes such as sE-selectin, sP-selectin and sICAM-1 [29-31]. Furthermore parasitized red blood cells have a stronger tendency to form rosettes with uninfected erythrocytes of the A, B or AB blood groups than with those of blood group O [32-36]. Rosette formation, however, does not occur with the infected erythrocytes specifically associated with placental malaria [35].

## Conclusion

In the meta-analysis of four studies a statistically significant reduction of placental malaria was observed for multiparous women with blood group O. For primiparous women no such effect was seen. The blood group O survival advantage with respect to the threat malaria poses is probably rather due to a milder course of the disease than due to a lower risk of infection, although the data from multiparous women suggest that a lower risk of infection may also play a role.

## Competing interests

The authors declare that they have no competing interests.

## Authors' contributions

AAA organised and led the day-to-day management of the study and contributed to the writing of the manuscript. AJFL, MPF, MY and PGK planned the study and contributed to the writing of the manuscript. MR supported the day-to-day management of the study and contributed to the writing of the manuscript. NGS supported the day-to-day management of the study, developed the analysis protocol, analysed the data and guided the writing of the manuscript. All authors contributed to the preparation of the manuscript and have approved the final version.

## Supplementary Material

Additional File 1 **Table S1: Studies on the association between ABO blood groups and placental malaria**. Click here for file
