# Supplementary material for: ABO blood group and the risk of placental malaria in sub-Saharan Africa
Source: Malar J. 2011 Apr 22;10:101. doi: 10.1186/1475-2875-10-101 (PMC3098819; doi:10.1186/1475-2875-10-101)
Supplement: Additional File 1 — Table S1: Studies on the association between ABO blood groups and placental malaria. [file 1475-2875-10-101-S1.DOC]

**Table S1: Studies on the association between ABO blood groups and placental malaria**

|  | **Loscertales et al.** | **Senga et al.** | **Adam et al. Malaria J.** (study listed here, but not included into Forest plot as meta analysis focused on holoendemic settings | **Adam et al. J. Parasit** (study only listed here, not included into comparisons) | **Present study** |
| --- | --- | --- | --- | --- | --- |
| Study period | 09/1967-05/1968 | 02-06/04, 01-07/05 | 10/06-03/07 | 11/07-01/08 |  |
| Setting | Banjul and Kombo-St Mary District of Gam­bia, seasonal malaria transmis­sion, peak after rainy season ( July- Oct.) | Chikwawa District, Southern Malawi, highly endemic perennial malaria transmission. | New Halfa teaching Hospital, Eastern Sudan. Low malaria transmission | Gadarif Hospital, eastern Sudan  Unstable malaria transmission | Lambaréné/ Gabon, perennial malaria transmission, EOR ≈50 infective bites per person year at risk. |
| Declared objectives | To describe the association between AB0 phenotypes, placental malaria and pregnancy outcomes | To confirm associations between AB0 phenotypes, placental malaria and pregnancy outcomes | To investigate the epidemio-pathological characteristics of placental malaria with special attention to blood-groups. | To determine the prevalence, and eval­uate the risk fac­tors, of placental malaria | To investigate the association between the ABO blood groups and active placental malaria |
| Analysis population | 198 mothers, 89 (45%) primiparae, active placental malaria in 74 (37%) | 647 mothers, 206 (32%) primiparae, active placental malaria in 124 (19%) | 293 mothers, 114 (40%) primiparae, active placental malaria in 12 (4%), past placental infection in 82 (, 28%) | 236 mothers, 111 (47%) primiparae, active placental malaria in 18 (7.6%), past placental in­fection in 28 (11.9%) | 378 mothers, 84 (22%) primiparae, active placental |
| Main exposure | ABO-blood group 0 vs. other | ABO-blood group 0 vs. other | ABO-blood group 0 vs. other | “Risk factors” for placental malaria | ABO-blood group 0 vs. other |
| Main Outcome | Active placental malaria | Active placental malaria | Past placental malaria (for the comparison here active placental malaria was used) | Past, acute & chronic placental malaria (for the comparison here active placental malaria was used) | Active placental malaria |
| Findings | Blood group 0 associated with  - a lower risk of active placental malaria infection in multiparae (OR 0.5, 95% CI 0.2-1.0)  - a higher risk of active placental malaria infection in primiparae (OR 3.0, 95% CI 1.2-7.3) | Blood group 0 associated with  - a lower risk of active placental malaria infection in multiparae (OR 0.6, 95% CI 0.4-1.0)  - a higher risk of active placental malaria infection in primiparae (OR 2.2, 95% CI 1.2-4.6) | Blood group 0 associated with  - a higher risk of past placental malaria infection in all (OR 1.9, 95% CI 1.1-3.2).  - a higher risk of past placental malaria infection in primiparae (OR 2.6, 95% CI 1.1-6.5). | No significant associ­ation between Blood group 0 and placental in­fection (OR 0.8, 95% CI 0.4-1.1). Main risk factor for placental infection was lack of prenatal care | Blood group 0 (non-significantly) associated with a higher risk of active placental malaria infection independently of the parity status (Combined OR 0.6, 95% CI 0.3-1.3) |
